# Mixed epithelial and mesenchymal metaplastic carcinoma (carcinosarcoma) of the breast: a case report

**DOI:** 10.1186/2047-783X-19-14

**Published:** 2014-03-13

**Authors:** Yu Kang, Shu Kang, Qingchang Li, Xinyu Zheng

**Affiliations:** 1 Department of Breast Surgery, the First Affiliated Hospital, China Medical University, North Nanjing Street 155, Shenyang 110001, P.R. China; 2 Department of Ultrasonography, the First Affiliated Hospital, China Medical University, North Nanjing Street 155, Shenyang 110001, China; 3 Department of Pathology, the First Affiliated Hospital and College of Basic Medical Sciences, China Medical University, North Nanjing Street 155, Shenyang 110001, China; 4 Lab 1, Cancer Institute, China Medical University, North Nanjing Street 155, Shenyang 110001, China

**Keywords:** metaplastic breast cancer, carcinosarcoma, treatment, progonosis

## Abstract

Metaplastic breast carcinoma (MBC) is an uncommon malignancy characterized by the co-existence of two or more cellular types, commonly a mixture of epithelial and mesenchymal components. A case of a female patient aged 46 years with MBC (carcinosarcoma) is presented, including mammographic, ultrasonic, gross examination, and pathological findings. After undergoing modified radical mastectomy of the left breast and subsequent six courses of adjuvant chemotherapy and endocrine therapy, the patient is now doing well with no recurrence and metastasis. Conventional treatments for invasive ductal carcinoma (IDC) may appear to be less effective. Patients with MBC would be appropriate candidates for innovative or targeted therapy regimens.

## Background

Metaplastic breast carcinoma (MBC) is a rare and heterogeneous group of malignancies that constitutes less than 1% of all breast cancers [1-3]. The World Health Organization recognized and classified metaplastic carcinoma in 2003 [4]. We report our experience with a case of a 46-year-old female who had a mixed epithelial and mesenchymal metaplastic carcinoma (carcinosarcoma) of the left breast, and we also present here a short review of the literature.

## Case presentation

A 46-year-old woman with two lumps in the upper outer quadrant of the left breast was referred to our department. The patient complained that the painless lumps have been growing rapidly over the previous three weeks. She had no history of trauma, nipple discharge, or other previous breast diseases. There was no known family history of breast cancer.

On clinical examination, palpation revealed two firm and mobile lumps closely adjacent to each other, measuring about 4.0 × 4.0 cm and 3.0 × 3.0 cm, respectively. There was no dimpling or puckering of the skin or changes of the skin color and the nipple. Axillary lymph nodes and other superficial lymph nodes were not palpable. Contralateral breast and axilla were normal.

**Figure 1:**
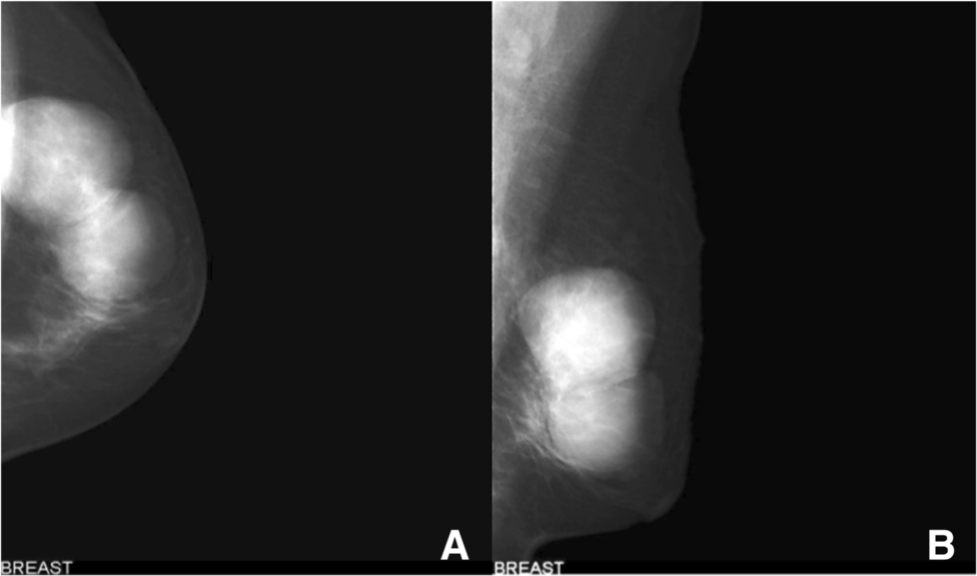
**Mammograms demonstrate two high-density masses. A**. Left craniocaudal mammogram shows two well-circumscribed round masses with internal heterogeneous micro-calcifications. **B**. Left mediolateral oblique mammogram shows two high-density masses in the upper outer quadrant of the left breast.

**Figure 2:**
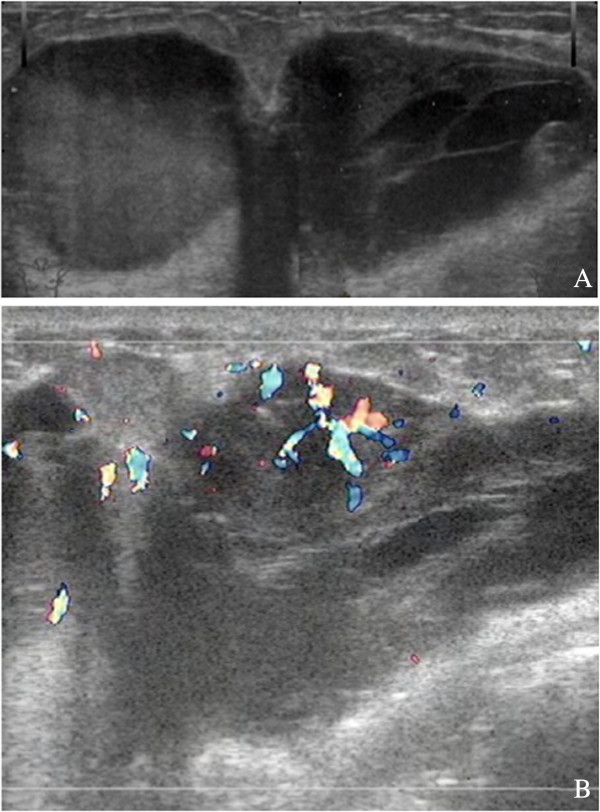
**A 46-year-old woman with palpable masses in the left breast. A**. Ultrasound demonstrates a pear-shaped complex echoic lesion with relatively indistinct margins. **B**. A hyperechoic area with abundant vessels within the mass. Another mass of the lesion is hyperechoic with spotted blood flow.

**Figure 3:**
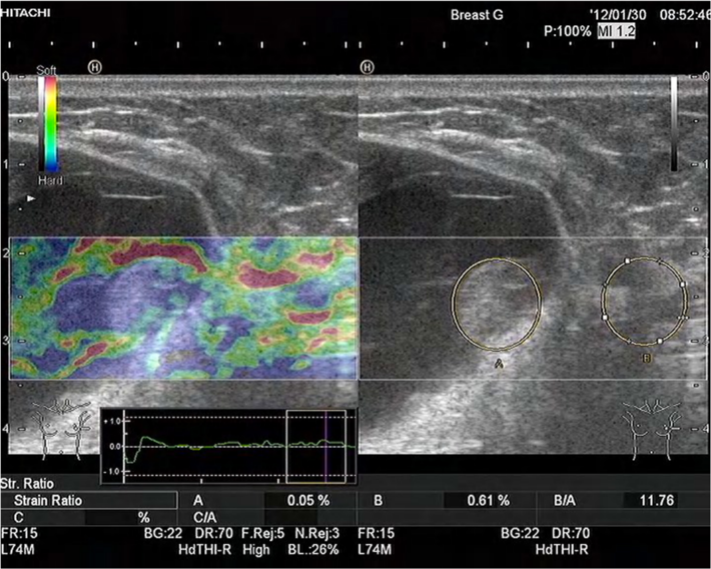
Left breast elastography shows the whole lesion was blue colored.

**Figure 4:**
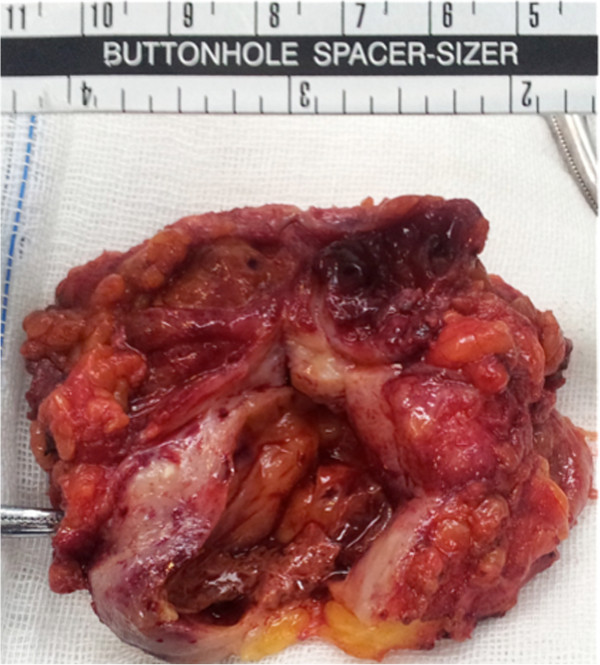
**Gross examination shows a cystic-solid tumor, which consisted of two parts.** Dark-red hemorrhage is noted.

**Figure 5:**
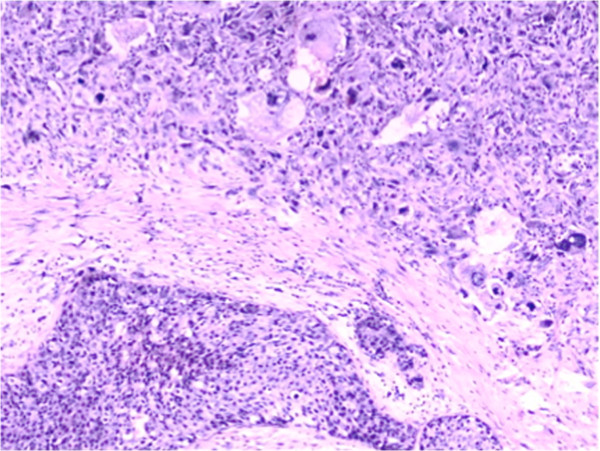
Epithelial and mesenchymal components are shown together (hematoxylin and eosin (HE) ×20).

Mammography revealed two well-circumscribed, round masses in the upper outer quadrant of the left breast, measuring 4.7 × 4.5 cm and 4.2 × 3.8 cm, respectively. The masses were generally high density, accompanied by heterogeneous micro-calcifications. The lesion corresponded to category 4B according to the BI-RADS Mammography lexicon classification [5] (Figure 1). Ultrasound demonstrated a pear-shaped, complex echoic lesion measuring approximately 8.3 × 3.7 × 7.3 cm with relatively indistinct margins in the upper outer quadrant of the left breast. One hypoechoic mass at the 2 o’clock position of the lesion was accompanied by a hyperechoic area with abundant vessels within the mass. The 3 o’clock mass of the lesion was hyperechoic with spotted blood flow. No enlarged lymph nodes were detected. An ultrasound diagnosis of intraductal papilloma accompanied by hemorrhage and solid mass (BI-RADS 4) [5] was made (Figure 2). The whole lesion was blue colored, with an elasticity score of 4 (Figure 3). Though no lymph nodes were detected in either clinical or ultrasonic examination, the patient did not agree with substituting axillary lymph node dissection (ALND) by sentinel lymph node biopsy (SLNB). Thus, modified radical mastectomy with ALND was performed. Gross examination of the specimen revealed a cystic-solid tumor with complete envelope, which consisted of two parts. The tumor was measured 7.0 cm in length and width, and 5.0 cm in height with the solid part measured 4.0 × 4.0 × 3.5 cm. Dark-red intracystic hemorrhage was noted (Figure 4). Microscopically, the tumor exhibited a biphasic pattern consisting of epithelial and mesenchymal components. Intraductal cell masses were formed in the epithelial components with obvious heteromorphism and central necrosis. Meanwhile, a large number of spindle cells with some multinucleate giant cells were present in the mesenchymal components in an interlaced pattern (Figure 5). Pathological diagnosis was mixed epithelial and mesenchymal metaplastic carcinoma (carcinosarcoma, histological grade III). No metastasis was found in 15 lymph nodes.

Immunohistochemically (images not included), the breast tumor showed negative for Cytokeratin (CK, CK5/6, and CK7), Desmin, S-100, and CD34 but showed positive for Vimentin, SMA, and CD68. However, the epithelial components expressed positive estrogen receptor (ER), progesterone receptor (PR), and C-erbB-2. In contrast, estrogen and progesterone receptors were negative, with weakly positive C-erbB-2, in the mesenchymal components. Ki-67 expression was 20%.

Having undergone three cycles of CEF (cyclophosphamide, epirubicin, and fluorouracil) and three cycles of T (Taxotere) followed by Tamoxifen, the patient is doing well one year after surgery with no recurrence and metastasis.

## Discussion

Accounting for less than 1% of breast cancers diagnosed annually [1-3], metaplastic breast carcinoma (MBC) is known to be characterized by the presence of two or more cellular elements histologically, commonly a mixture of epithelial and mesenchymal components [6-10]. Wargotz and Norris suggested that carcinosarcoma of the breast, in which an epithelial-mesenchymal transition zone does not exist, should be distinguished from other MBC diseases. Such diagnosis is not difficult with detailed histological investigation [7]. Classification of metaplastic carcinoma was proposed by the World Health Organization in 2003 as 1) squamous cell carcinoma, 2) adenocarcinoma with spindle cell proliferation, 3) adenosquamous, including mucoepidermoid, and 4) mixed epithelial and mesenchymal. Subtypes of mixed epithelial and mesenchymal carcinoma includes a) carcinoma with chondroid metaplasia, b) carcinoma with osseous metaplasia, and c) carcinosarcoma [4]. Carcinosarcoma is a general term describing biphasic lesions that simultaneously contain malignant epithelial and malignant mesenchymal tissue components [11]. The origin of breast carcinosarcoma is far from clear. They have been reported to develop from existing cystosarcoma phyllodes, fibroadenoma and cystic backgrounds [12-14]. Carcinosarcoma is characterized by the loss of intercellular adhesion, down-regulation of epithelial makers (cytokeratins), upregulation of mesenchymal markers [vimentin and smooth muscle actin (SMA)], increase in motility, invasiveness, and metastatic capabilities [15-19].

Although relatively rare and histologically heterogeneous, their clinical manifestations are often similar. They are often palpable, mobile, and large, showing benign imaging features such as a round or oval shape with circumscribed margins. Meanwhile, estrogen, progesterone, and Her-2 are less frequently shown to be positive, with lower rates of axillary node involvement [20-24]. But approximately 70% of metaplastic carcinomas show epidermal growth factor receptor (EGFR) gene amplification and overexpression [25].

The optimal treatment strategies for MBC are unknown. Currently, management of MBC has largely paralleled that of invasive ductal carcinoma (IDC). Theoretically, MBC patients should undergo mastectomy rather than lumpectomy due to larger masses compared to their IDC counterparts. However, studies have found no difference in overall or disease-free survival between patients with MBC undergoing either modified radical mastectomy or breast conservation therapy [26,27]. Traditional adjuvant chemotherapy for IDC is ineffective against MBC [28-30], so is hormonal therapy as there is a high incidence of hormone receptor negativity in MBC [31]. Tseng *et al*. suggested that adjuvant radiation improved both overall and disease-specific survival for all patients undergoing treatment for MBC, regardless of the type of operation performed (lumpectomy versus mastectomy) [26]. Treatment given in the neoadjuvant setting has become the standard approach for potentially operable breast carcinomas with benefits including tumor downsizing, earlier treatment of micrometastatic disease, and the ability to assess responsiveness to therapy directly. However, it is important to identify patients who would benefit from this approach and those who would not. We should be cautious when considering neoadjuvant chemotherapy given to those with MBC because several studies indicated that these patients responded poorly and might gain less benefit from standard regimens [20,32,33]. In addition, samples obtained by either fine needle aspiration or core needle biopsy might not be sufficient to distinguish MBC from common types of breast cancer, which suggests that it could be difficult to make an accurate diagnosis preoperatively [21,34,35]. Therefore, conventional neoadjuvant regimens, if given to patients with MBC, may carry the risks of tumor progression. Takuwa *et al.* reported a case of MBC that responded well to platinum-based neoadjuvant chemotherapy, resulting in nearly a pathologically complete response [36]. Nevertheless, careful monitoring is essential since the failure of chemotherapy may result in clinical deterioration and preoperative complications. Under the circumstance of ineffectiveness of conventional therapies, innovative or targeted treatments are being explored. Since MBC tends to be EGFR positive while Her-2 tends to be negative, Leibl and Moinfar suggested that targeted protein kinase inhibitors such as gefitinib might be effective [25].

The prognosis of MBC still remains controversial. Some studies reported that compared to patients with IDC, those with MBC have a worse prognosis. Their overall survival and disease-free survival are both decreased despite presenting more commonly as node-negative disease [37,38]. Conversely, other studies showed comparable outcomes with matched typical breast cancer [2,21]; however, almost all MBC recurrences happened during the first five years, whereas recurrence curves for IDC continued to fall over time, suggesting the possibility that MBC may show an earlier recurrence than IDC [21].

## Conclusions

Generally, MBC is often palpable, mobile, and large, showing a round or oval-shaped mass with a circumscribed margin in the mammogram. Given the heterogeneity of MBC, an accurate preoperative diagnosis may not be achieved with needle biopsies. Conventional treatments for IDC also appear to be less effective. Patients with MBC would be appropriate candidates for innovative or targeted therapy regimens. Prospective studies are needed, but the rarity of MBC makes these less likely to be conducted.

## Consent

Written informed consent was obtained from the patient for publication of this case report and any accompanying images. A copy of the written consent is available for review by the Editor-in-Chief of this journal.

## Abbreviations

ALND: axillary lymph node dissection; HE: hematoxylin and eosin stain; ER: estrogen receptor; IDC: invasive ductal carcinoma; MBC: metaplastic breast carcinoma; PR: progesterone receptor; SLNB: sentinel lymph node biopsy.

## Competing interests

The authors declare that they have no competing interests.

## Authors’ contributions

YK drafted the manuscript and revised it. SK, QCL, and XYZ were responsible for images and corresponding interpretations. XYZ contributed to manuscript proofreading and revisions. All authors read and approved the final manuscript.
